# Iron and Alzheimer’s Disease: From Pathogenesis to Therapeutic Implications

**DOI:** 10.3389/fnins.2018.00632

**Published:** 2018-09-10

**Authors:** Jun-Lin Liu, Yong-Gang Fan, Zheng-Sheng Yang, Zhan-You Wang, Chuang Guo

**Affiliations:** ^1^College of Life and Health Sciences, Northeastern University, Shenyang, China; ^2^Department of Dermatology, First Hospital of Qinhuangdao, Qinhuangdao, China; ^3^Key Laboratory of Medical Cell Biology of Ministry of Education, Institute of Health Sciences, China Medical University, Shenyang, China

**Keywords:** Alzheimer’s disease, iron, chelation, alpha-lipoic acid, lactoferrin

## Abstract

As people age, iron deposits in different areas of the brain may impair normal cognitive function and behavior. Abnormal iron metabolism generates hydroxyl radicals through the Fenton reaction, triggers oxidative stress reactions, damages cell lipids, protein and DNA structure and function, and ultimately leads to cell death. There is an imbalance in iron homeostasis in Alzheimer’s disease (AD). Excessive iron contributes to the deposition of β-amyloid and the formation of neurofibrillary tangles, which in turn, promotes the development of AD. Therefore, iron-targeted therapeutic strategies have become a new direction. Iron chelators, such as desferoxamine, deferiprone, deferasirox, and clioquinol, have received a great deal of attention and have obtained good results in scientific experiments and some clinical trials. Given the limitations and side effects of the long-term application of traditional iron chelators, alpha-lipoic acid and lactoferrin, as self-synthesized naturally small molecules, have shown very intriguing biological activities in blocking Aβ-aggregation, tauopathy and neuronal damage. Despite a lack of evidence for any clinical benefits, the conjecture that therapeutic chelation, with a special focus on iron ions, is a valuable approach for treating AD remains widespread.

## Introduction

Alzheimer’s disease (AD) is a neurodegenerative disease that occurs in the elderly population. Most patients show early loss of memory, and as the condition worsens, language disorders, loss of directionality, and anxiety behaviors will also be present (Cheng et al., 2013). Regarding late-stage patients, their mental activities, such as cognition, emotion and behavior, are abnormal, and their bodily functions are gradually lost (Ikonomovic et al., 2011). With the development of society and changes in the human environment, the incidence of AD has increased year by year. In the epidemiological survey performed by the Alzheimer’s Association in the United States in 2017, the number of AD patients in the United States exceeded 5.5 million, and people over the age of 65 were found to be twice as likely to suffer from AD. However, the pathogenesis of AD remains unclear, and drugs that can completely cure AD or relieve symptoms have not yet been developed (Blennow et al., 2006; Huang and Mucke, 2012; Mullard, 2012; Reese et al., 2012; Selkoe, 2012).

## The Pathogenic Hypothesis of Alzheimer’S Disease

The two major histopathological features of AD in the brain are senile plaques (SPs), formed by the deposition of extracellular β-amyloid protein (Aβ), and neurofibrillary tangles (NFTs), formed by hyperphosphorylation of tau proteins associated with microtubules in neurons (Colvez et al., 2002). Based on these obvious pathological features, scholars have proposed two hypotheses about the developmental mechanism of AD: the amyloid cascade hypothesis and the NFTs hypothesis. With the deepening of research, more recent scholars also proposed the hypothesis of inflammation and the metal ion hypothesis and continuously enriched the developmental mechanism of AD.

The amyloid cascade hypothesis stems from the amyloid degradation pathway of β-amyloid precursor protein (APP). This hypothesis states that Aβ1-42, produced by the amyloid degradation pathway of APP, has significant neurotoxicity, can induce aggregation and hyperphosphorylation of tau protein, form NFTs, cause neuronal damage, and eventually lead to dementia (Klein et al., 2001). The NFTs hypothesis originates from the presence of a large number of fiber tangles formed by the aggregation of hyperphosphorylated tau proteins in the neurons of AD patients. This hypothesis suggests that hyperphosphorylated tau protein competes with normal tau protein for binding to tubulin, disrupting the dynamic balance of microtubule assembly and disaggregation (Cardenas-Aguayo Mdel et al., 2014) and resulting in impaired axonal transport and accumulation of intracellular waste. Neurons gradually degenerate and cause dementia. In addition to amyloid plaques and NFTs, the researchers found a large amount of activated astrocytes and microglia in the brains of AD patients, accompanied by increased expression levels of TNF-α, IL-1β, IL-6 and other inflammatory factors (Cunningham et al., 2005). Therefore, the neuroinflammation hypothesis was proposed. This hypothesis suggests that neuroinflammation is not a passive system in AD that is activated by SPs and NFTs, but, like plaques and tangles, plays an important role in the development of diseases (Zhang et al., 2013).

Interestingly, substantial evidence has indicated that the steady-state dysregulation of metal ion metabolism *in vivo* can be involved in the pathology of AD (Kim et al., 2018). An imbalance in the metal levels in the brains of AD patients has been identified and is accompanied by metal-catalyzed oxidative damage (Nunomura et al., 2001; Perry et al., 2003). A large number of studies have shown that metal ions, such as copper, iron, zinc, magnesium and aluminum, are involved in the occurrence and development of AD (Wang and Wang, 2017). Clinical studies have also shown elevated levels of copper, iron and zinc in the brains of AD patients (Bush, 2013). Metal ions can affect neuronal metabolism, cause oxidative stress, and promote the deposition of Aβ and the formation of SPs (Lovell et al., 1998). At the same time, studies have also shown that the deposition of Aβ in the brain and its toxicity are directly related to metabolic disorders of zinc, copper, iron, and other metal ions in the cortex and the hippocampus (Liu et al., 2006). Studies have also shown that an imbalance of metal homeostasis can directly cause neuronal dysfunction (Myhre et al., 2013) and lead to neuronal cell death (Wright and Baccarelli, 2007). Moreover, the successful application of metal (zinc, copper, iron) chelators in several animal models of AD and patients with early AD provided strong evidence that AD is a transition metal-overloading disease (Guo et al., 2013b, 2015, 2017; Dusek et al., 2016; Giampietro et al., 2018; Kawahara et al., 2018; Zhang et al., 2018). Based on the research described above, the metal ion hypothesis was proposed to emphasize the role of metal ions in the pathogenesis of AD, which further complemented the pathogenesis of AD.

## Brain Iron Dyshomeostasis and the Pathophysiology of Alzheimer’S Disease

Iron is the second most abundant metal on earth, second only to aluminum, and it is also an essential element for the survival of all living things on earth (Crielaard et al., 2017). The biological activity of iron depends, to a large extent, on its effective electron transfer properties, allowing it to accept or provide electrons during the transition between divalent, ferric and tetravalent iron states of ferrous iron, thus serving as a catalytic cofactor in a variety of biochemical reactions (Hohenberger et al., 2012). Iron also promotes the activity of various biological enzymes in the process of DNA replication and repair in the form of iron–sulfur clusters (Fe-S). Simultaneously, iron is also a component of hemoglobin and myoglobin, which is involved in the transport of oxygen and carbon dioxide in organisms (Takeda, 2004).

Many important physiological activities in the brain involve iron. If iron is absent during the development of the brain, it will cause irreversible developmental delays; however, if the iron overload in the brain also has a neurotoxic effect, this will damage the normal physiological activities of the brain. The iron content in the brain gradually increases with age. Interestingly, using magnetic resonance imaging (MRI), it was found that the iron content in the brains of AD patients was significantly increased (Du et al., 2018). This finding was also confirmed in a comparative study of APP/PS1E9 double transgenic AD mice and wild-type mice of the same age (Dwyer et al., 2009). Besides, some scholars believe that an iron metabolism disorder is an important cause of late-onset AD formation (Corder et al., 1995). Based on the various discoveries related to iron, researchers realized that iron plays an extremely important role in the occurrence of AD. Therefore, research on iron as a target has gradually become a new direction for scientists to explore the pathogenesis of AD.

### Iron Absorption and Transport Into the Brain

Iron mainly exists in non-heme iron and heme iron in foods, and non-heme iron accounts for 90% of them. Non-heme iron is reduced to Fe^2+^ in the upper part of the small intestine and enters mucosal epithelial cells via a divalent metal ion transporter (DMT1) on the membrane of the small intestine epithelium. The remaining 10% of heme iron reacts with heme oxygenase (HO) in the proximal part of the small intestine, releasing Fe^2+^ and is directly taken up by small intestinal mucosa epithelial cells via DMT1 (Krishnamurthy et al., 2007; Horl, 2008). Fe^2+^ uptake by epithelial cells can be used directly by cells, while unutilized Fe^2+^ is oxidized by ferrous oxidase (hephaestin) or ceruloplasmin to Fe^3+^. The resulting Fe^3+^ is transported out of the cell via the ferroportin 1 (FPN1) of the basolateral membrane of the intestinal mucosal epithelium. The transferred out Fe^3+^ is mainly combined with the transferrin (Tf) in the blood to form the iron-transferrin complex and can also be combined with lactoferrin to form a non-transferrin-bound iron into the peripheral blood circulation (Frazer and Anderson, 2005; Garrick and Garrick, 2009).

**FIGURE 1:**
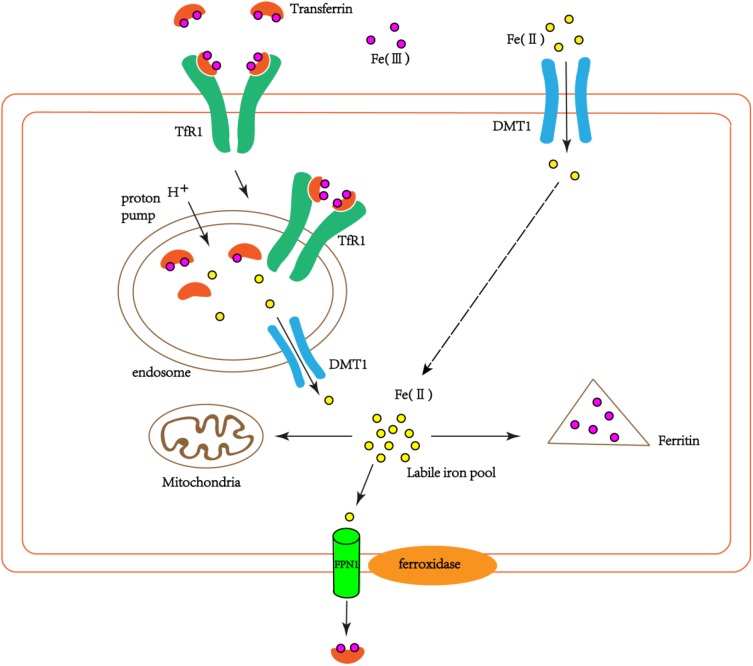
The schematic diagram of iron transport into cells. Part of the extracellular bivalent iron can be directly transferred into cells through DMT1. Transferrin-bound iron binds TfR1 through endocytosis to form endosomes in the cells. Due to the action of the proton pump on the endosome, trivalent iron dissociates from the Tf/TfR1 complex and is reduced to divalent iron, which enters the cytoplasm via DMT1. Part of the ferrous iron that enters the cytoplasm is used by the cell itself (such as mitochondria), and part of it is oxidized to ferrous iron by ferritin and stored. Another part is oxidized to ferric iron by the ferroxidase on the cell membrane, and the cells are exported by FPN1 and recombined with extracellular Tf.

The iron-transferrin complex circulating in the peripheral blood to the brain enters the cells through endocytosis of brain capillary endothelial cells. This endocytosis is mediated mainly by transferrin receptor (TfR) on the surface of endothelial cells. Non-transferrin-bound iron in peripheral blood can enter the brain through the lactoferrin/lactoferrin receptor pathway (Ke and Qian, 2007). Iron-transferrin complexes form endosomes into cells via endocytosis. Due to the action of the proton pump on the endosome membrane, the PH in the endosome is reduced, which leads to the dissociation of iron and Tf/TfR complexes, at the same time Fe^3+^ is reduced to Fe^2+^. Fe^2+^ enters the endothelial cytoplasm through DMT1 on the endosome membrane (**Figure 1**). The detached iron Tf/TfR complex exudes from the vesicle to the lateral lumen of the endothelial cells. In the pH 7.4 environment, Tf dissociates from TfR and re-enters the blood (Dringen et al., 2007).

### Iron Regulation in the Brain

According to autopsy reports, total iron deposition in the human brain is positively related to age and contains high concentrations of iron in the basal ganglia of the putamen, globus pallidus, and substantia nigra (Connor et al., 1995), whereas the cerebral cortex, the brainstem and cerebellum contain low concentrations of iron (Zecca et al., 2004; Ramos et al., 2014). Iron homeostasis in nerve cells is mainly regulated by the transcriptional levels of mRNAs involved in iron metabolism. The proteins involved in brain iron metabolism mainly include iron regulatory proteins (IRPs), Tf, TfR1, ferritin, FPN1, DMT1, and so on (Crielaard et al., 2017) The mRNAs encoding TfR1, ferritin, FPN1 and DMT1 all contain a special amino acid sequence called the iron regulatory element (IRE). Iron regulates the transcription of iron-related proteins by controlling the binding of IRPs to IRE, thereby maintaining intracellular iron homeostasis (Zhou and Tan, 2017). After continuous in-depth research, people further realized that hepcidin, an antibacterial peptide, is an important factor in iron homeostasis, particularly brain iron homeostasis (Vela, 2018). FPN1 is the major receptor for hepcidin *in vivo*. A series of studies demonstrated that hepcidin regulates iron homeostasis via direct interaction between hepcidin and FPN1, inducing the internalization and degradation of FPN1 reducing the ability of cells to export iron (De Domenico et al., 2008), and thereby increasing the possibility of intracellular iron overload (Daher et al., 2017). Later, cell experiments showed that hepcidin not only downregulated the expression of FPN1 in astrocytes and neurons but also downregulated the expression of TfR and DMT1 (Du et al., 2011, 2015). The above results suggested that hepcidin regulates iron homeostasis not only by controlling the iron output of cells but also by regulating the iron input of cells.

In addition to iron-related proteins that regulate iron, APP and tau also act to regulate iron. Studies have shown that APP is a certain regulator for iron homeostasis, which can interact with FPN1 to regulate the efflux of ferrous ions (Kawahara et al., 2017). Indeed, APP knockout or haplo-insufficiency preferentially mediates brain iron accumulation in mice (Duce et al., 2010). Tau acts as an intracellular microtubule-associated protein, which can transport the produced APP to the cell surface to promote iron output (Li et al., 2015). Interestingly, tau knockout mice develop age-dependent iron accumulation and brain atrophy, and iron retention in the primary cultured neurons is caused by decreasing surface trafficking of APP, indicating that tau-mediated iron homeostasis might be APP-dependent(Lei et al., 2012; Tuo et al., 2017).

### Iron Participates in the Occurrence of Alzheimer’s Disease

In the brain, iron is not only involved in the synthesis of myelin and neurotransmitter synthesis and metabolism but also plays an important role in maintaining the high metabolic capacity of neurons (Gerlach et al., 1994). Under normal physiological conditions, iron metabolism maintains homeostasis in the brain. Once iron metabolism is out of balance, it will have different effects on brain function. As early as Goodman (1953) found that iron was increased in the SPs in the brains of AD patients. Later, the use of quantitative susceptibility map (QSM) once again confirmed the co-localization of brain iron and Aβ plaques, and showed that the co-localization of brain iron deposition and Aβ plaques promoted the development of the disease (van Bergen et al., 2016). In fact, there are also progressive iron deposits that occur in the normal aging process of the brain, particularly in the substantia nigra, globus pallidus, caudate nucleus, and cortex, and these brain regions are closely related to neurodegenerative diseases (Rodrigue et al., 2011; Callaghan et al., 2014; Collingwood and Davidson, 2014; Ward et al., 2014). Compared with healthy people of the same age, the iron deposition in patients with AD is more serious in these areas. Further, the APP mRNA of peripheral blood mononuclear cells of AD patients was significantly lower than that of the control group by fluorescence quantitative PCR, which indirectly indicated the iron dyshomeostasis in AD (Guerreiro et al., 2015).

#### Iron Participates in the Deposition of Aβ Plaques and Tau Tangles

Studies have demonstrated that iron metabolic disorders can induce the production and accumulation of Aβ because iron can act on the IRE site of APP mRNA, thereby enhancing the translation and expression of endogenous APP (Cahill et al., 2009). It has also been found that long-term administration of high concentrations of iron in APP/PS1E9 double transgenic mice results in an increase in the number of SPs in the brain (Smith et al., 1997). When extended iron exposure to 12 months, the increased brain iron with 3,5,5-trimethylhexanoyl ferrocene diet accelerated the formation of SPs and microglial iron inclusions in APP mice (Peters et al., 2018).

Although there is a lot of evidence that iron and Aβ plaques co-localize, it is not known what form iron is present in plaques. Recently, Plascencia-Villa et al. (2016) used transmission electron microscopy (TEM) to confirm that iron is present in the core of SPs in the form of iron oxide (Fe_3_O_4_) magnetite nanoparticles. This provides evidence of metal biology associated with iron accumulation and Aβ aggregation. Later, using *in situ* X-ray magnetic circular dichroism again revealed the existence of magnetite in human SPs (Everett et al., 2018). Magnetite, as a polycrystalline iron oxide, is not a normal feature in the human brain, and its elevated content indicates that the anomalous iron redox chemistry affects AD (Ayton et al., 2017b). Furthermore, Telling et al. (2017) used advanced sub-microscopic resolution of X-ray microscopy to find evidence of the direct correlation between the morphology of SPs and iron and the formation of iron-amyloid complexes. Importantly, Aβ binds to iron through three histidine residues and one tyrosine residue in the hydrophilic N-terminal region of the peptide, which helps to stabilize these iron ions (Lane et al., 2018). In turn, studies also found that the binding of ferrous ions to Aβ reduced the peptide helix structure and increased the β-sheet content of the peptide, indicating that ferrous ions promote amyloid monomers to form oligomers and fibrils by enhancing the interaction between peptide-peptides (Boopathi and Kolandaivel, 2016; Tahirbegi et al., 2016). Except promoting Aβ aggregation, high iron levels can impact amyloidogenic processing of APP. Early studies found that iron had a modulatory effect on the α-secretase cleavage activity of APP (Bodovitz et al., 1995). Later, studies found that the process of converting α-secretase and β-secretase from the inactive state to the active state was regulated by furin, and iron could regulate the expression of furin at the transcriptional level (Silvestri and Camaschella, 2008). Excessive iron inhibits the expression of furin, which favors the activation of β-secretase, thereby promoting the production of Aβ from the amyloid pathway (Ward et al., 2014). Another study found that presenilin enhancer 2 (PEN-2), a γ-secretase components, could bind to iron through the ferritin light chain, enhancing γ-secretase activity, thereby increasing Aβ formation (Li et al., 2013).

*In vitro* studies have also found that iron can promote aggregation of Aβ peptides and increase their cytotoxicity (Tahmasebinia and Emadi, 2017; Galante et al., 2018). However, there are different opinions on the role of iron and Aβ. Earlier studies have found that Fe^2+^ and Fe^3+^ interacted with APP and Aβ to promote aggregation of Aβ into fibrous forms (Ha et al., 2007). Fe^2+^ can also interact with the amino acids of the Aβ protein, which may impart changes in the form of amyloid in a different manner than copper and zinc (Dahms et al., 2012). Fe^3+^ bound to Aβ is easily reduced to Fe^2+^ and increases reactive oxygen species (ROS) production, which causes β-secretase to cleave monomer Aβ42 into more toxic Aβ oligomers, accelerating neuronal death (Cohen et al., 2013; Balejcikova et al., 2018). Importantly, Aβ can damage mitochondrial function, convert Fe^3+^ into Fe^2+^ with redox activity, and induce oxidative stress, thereby aggravating iron overload and aggravating the AD condition (Everett et al., 2014; Mena et al., 2015). Further, it has been shown that iron exposure promotes the accumulation of APP in cultured SHSY5Y cells, along with the increase of β-secretase activity and Aβ42 in the medium (Banerjee et al., 2014). Inconsistently, a recent study showed that iron treatment of neurons promoted the APP non-amyloid pathway, altered the distribution of sAPPα and retained it in cell lysates rather than secreted outside the cell, while iron did not change β-secretion enzyme expression, but significantly inhibits its activity (Chen et al., 2018). Another study also found that Aβ can significantly reduce the redox ability of iron, which may indicate neuroprotection and metal chelation of Aβ during the pathogenesis of AD, but becomes toxic under certain conditions (Lane et al., 2018).

**FIGURE 2:**
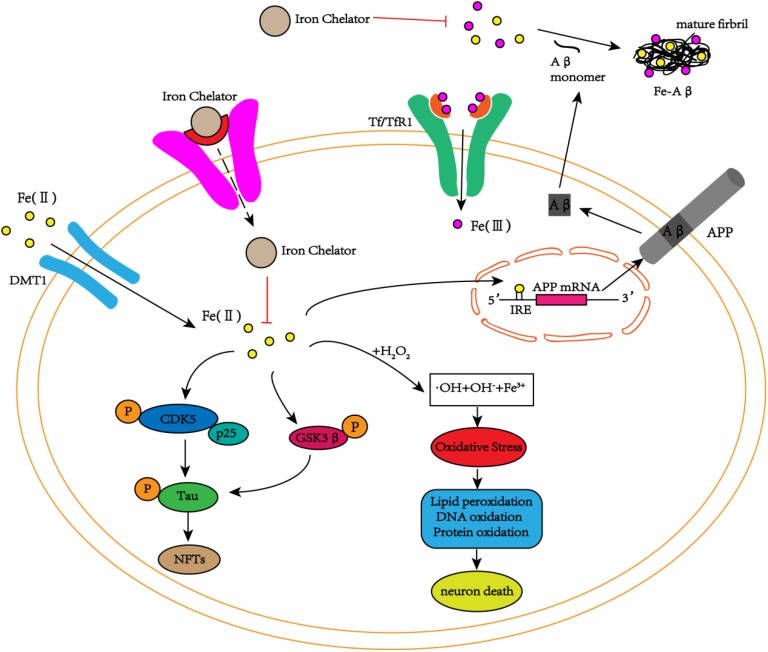
The schematic diagram of iron participation in the deposition of Aβ plaques and tau tangles. In neurons, iron interacts with Aβ and promotes Aβ aggregation into fibrous forms. Iron can also act on the IRE site of APP mRNA, increasing the expression of endogenous APP. In addition to the interaction with Aβ, iron can also promote the phosphorylation of tau by activating the CDK5/p25 complex and GSK3β to form NFTs. At the same time, iron can also cause oxidative stress through the Fenton reaction, damaging DNA, lipids and proteins and eventually leading to cell death. The iron chelators reduce the phosphorylation of tau and inhibit the production of NFTs by inhibiting the activation of the CDK5/p25 complex and GSK3β by iron. Simultaneously, iron chelators inhibit the aggregation of Aβ monomers into toxic fibrous forms by chelating iron, delaying cell death.

Neurofibrillary tangles are another major pathological feature of AD, and phosphorylated tau protein is the main component of NFTs. Studies have found iron deposition in neurons with NFTs (Smith et al., 1997). In addition to Aβ peptides, iron can bind to tau protein, induce tau protein phosphorylation, and aggregate phosphorylated tau protein, whereas this phenomenon can be reversed by iron chelators (Amit et al., 2008). Fe^3+^ induces the aggregation of hyperphosphorylated tau protein, and when Fe^3+^ is reduced to Fe^2+^, its induced aggregation can be reversed (Yamamoto et al., 2002). These results reveal that iron may play an important role in the accumulation of hyperphosphorylated tau protein to form NFTs. Recent studies have shown that tau protein indirectly participates in the transmission of iron ions in brain neurons during the pathogenesis of AD (Lei et al., 2012). Moreover, *in vitro* and *in vivo* experiments have shown that iron is involved in the hyperphosphorylation of tau protein through the activation of the cyclin-dependent kinase (CDK5)/P25 complex and glycogen synthase kinase-3β (GSK-3β) (Xie et al., 2012; Guo et al., 2013a), but no relevant experiments have reported whether iron is also related to the inactivation of the protein phosphatase PP2A (**Figure 2**). Additionally, Fe^3+^ can promote the reduction of superoxide radicals released by mitochondria (Kudin et al., 2004; Aliaga et al., 2011). The reduction of Fe^3+^ can lead to the production of superoxide, and the reaction of superoxide with nitric oxide (NO) to produce per-nitrate can damage tyrosine residues that function normally (Nakamura and Lipton, 2011). In AD, nitration of tau prevents its stabilization of the microtubule lattice, and nitrating tau protein has been observed in tau entanglement and SPs (Reynolds et al., 2006; Kummer et al., 2011). Interestingly, the accumulation of tau in NFTs is also associated with increased induction of HO1 (Wang et al., 2015). HO1 is a potent antioxidant that can metabolize heme released from damaged mitochondria, and it also promotes the release of Fe^2+^, which may cause free radicals to initiate additional oxidative stress (Ward et al., 2014). Thus, iron-induced oxidative stress may also promote hyperphosphorylation and aggregation of tau (Lane et al., 2018).

To date, although the specific mechanism of iron involvement in the aggregation of Aβ plaques and hyperphosphorylated tau protein is not yet clear, it has been shown that iron can accelerate this process by affecting related signaling pathways and the three-dimensional conformation of proteins. Nevertheless, the changes in iron levels in AD need to be emphasized, and homeostatic imbalance may have dual effects through iron induction. On the one hand, iron overload areas in the brain contribute to oxidative stress and cell death around SPs and NFTs; on the other hand, other areas of the brain may suffer from impaired neuronal function due to iron deficiency (Belaidi and Bush, 2016).

#### Ferroptosis and AD

Cell death plays an important role in the growth and development of organism and tissue homeostasis. Studies have found that cell death is dysregulated in AD (Yang and Stockwell, 2016). Based on the unique pathological state caused by iron overload, scholars have also proposed a fourth cell death mode that differs from apoptosis, necrosis, and autophagy, namely, ferroptosis (Dixon et al., 2012).

Ferroptosis refers to iron-dependent lipid peroxidation-induced cell death that depends on ROS production and iron availability, with severe lipid peroxidation (Dixon, 2017). One of the hallmarks of ferroptosis is the iron-dependent accumulation of lipid ROS, a form of death that is dependent on intracellular iron rather than other metals (Abdalkader et al., 2018). The morphological features of ferroptosis are mainly reflected in intracellular mitochondria. Compared to the mitochondria of normal cells, the mitochondrial volume of ferroptosis cells is smaller, the density of mitochondrial membranes is reduced, mitochondrial hemorrhoids are reduced or have disappeared, and the mitochondrial outer membrane ruptures (Xie et al., 2016). Moreover, studies have found that the occurrence of ferroptosis consists of the accumulation of lipid ROS triggered by the inactivation of the intracellular antioxidant glutathione (GSH). Therefore, ferroptosis is caused by the imbalance in cellular redox homeostasis (Gao et al., 2016). Glutathione peroxidase 4 (GPx4), an antioxidant defense enzyme that repairs oxidative damage to lipids, is a central endogenous suppressor of ferroptosis (Chen et al., 2015). Studies have found that the GPx4 gene knockout mice involve in both three pronounced hallmarks of ferroptosis (iron dysregulation, lipid peroxidation, inflammation) and prodromal indices of AD (behavior dysfunction, hippocampal neurodegeneration), and these pathological changes can be ameliorated or prevented by a ferroptosis inhibitor (Seiler et al., 2008; Hambright et al., 2017). Intriguingly, erastin, a ferroptosis attractant, can induce neuronal death accompanied by ferroptosis (Hirata et al., 2018). Conversely, iron chelators and antioxidants specifically involved in protecting cells against ferroptosis (Hambright et al., 2017). Taken together, although the physiological function of ferroptosis is still unclear, its role in age-related neurodegenerative diseases (including AD) has been established. This suggests that considering ferroptosis the center, the development of ferroptosis inhibitors may be a new direction to alleviate the symptoms of AD.

## Biomarkers for Clinical Diagnosis of Ad Progression

The pathogenesis of AD has not yet been fully explained. If special methods can be used to detect AD in the early stage, it is very important to increase our understanding of the disease and active prevention of the disease. Currently, the clinical diagnosis of AD is based primarily on family history and certain cognitive dysfunctions (Alzheimer’s, 2016). Other methods mainly detect the levels of Aβ1-42 and phosphorylated tau and total tau in cerebrospinal fluid to predict the development and severity of AD (Bulk et al., 2018). A common method for detecting Aβ plaques is positron emission tomography (PET). With the deepening of research, more and more new technologies are used to identify changes in the Alzheimer brain, and gradient echo multiple contrast imaging (GEPCI) technology is one of them. The study confirmed a strong correlation between the GEPCI brain tissue index and the Aβ load defined by PET, providing a new method for assessing AD-related histopathology in the preclinical and early symptom stages of AD (Zhao et al., 2017).

Although Aβ and tau degeneration are considered to be key factors in AD, iron dyshomeostasis is increasingly reported as a potential cause of AD pathophysiology. Can iron be used as a biomarker for the progression of AD to detect the occurrence of AD and reflect the severity of AD? For this reason, iron as a biomarker of AD has become a research hotspot for scientists. The current effective method for detecting brain iron levels is QSM. QSM has better specificity and can be used to non-invasively assess tissue magnetic susceptibility, which has been confirmed to have a good correlation with brain iron levels (Du et al., 2018). Through QSM, Du et al. (2018)found that the magnetic susceptibility values of the caudate nucleus on the left side of the brain may be used as biomarkers for the severity of mild and moderate AD disease. As early as Moon et al. (2016) have found that the magnetic susceptibility values of the core and caudate nucleus of AD patients are significantly different from those of the control group. Later, van Bergen et al. (2018) jointly used QSM and PET to show that the local correlation of Aβ plaque load and iron deposition can provide relevant information about the cognitive performance of healthy elderly people. Studies have shown that AD patients have higher iron accumulation in the frontal cortex, and the degree is related to the number of Aβ plaques and NFT, and there is a different iron distribution, and these changes appear to occur after the development of AD pathology markers (van Duijn et al., 2017). These findings suggest that changes in iron-based magnetic resonance (MRI) contrast can be used to indirectly determine the extent of AD pathology. Although iron concentration was identified as the main source of comparison in QSM in the brain, O’Callaghan et al. (2017) found a correlation between QSM and tau concentration, suggesting that QSM may be a useful biomarker for early detection of tau pathology in AD.

As a major iron storage protein in the body, ferritin is closely related to AD. Elevated ferritin in CSF has been shown to be associated with poor cognitive function and increases the risk of mild cognitive impairment to AD (Ayton et al., 2015). Moreover, research has shown ferritin’s potential to contribute to a blood biomarker panel for preclinical AD (Goozee et al., 2017). CSF ferritin levels were negatively correlated with cognitive performance and strongly correlated with apolipoprotein E (ApoE) in CSF, suggesting that CSF ferritin as an indicator of brain iron load may be a biomarker of AD cognitive function (Ayton et al., 2015). However, another study combining QSM and PET to predict the value of longitudinal cognitive deterioration found that iron can worsen cognitive function in the presence of Aβ pathology. There is no Aβ pathology, and iron has no correlation with cognitive function (Ayton et al., 2017a). More advanced, Ayton et al. (2018) further found that high concentrations of CSF ferritin can accelerate the decrease of Aβ in CSF of 296 participants, which supports the possibility that iron may promote Aβ deposition and accelerate disease progression. This is the first clinical evidence that iron is associated with amyloid plaque formation.

Due to the invasiveness of lumbar puncture, the use of this CSF biomarker limits the widespread clinical application, while serum or plasma biomarkers are relatively simple to acquire and less invasive, and thus have great potential for application in AD. Studies have shown that serum and iron-related protein levels in AD patients are significantly elevated (Sternberg et al., 2017). Unlike Aβ peptides, iron and iron-related proteins are significantly associated with cognitive assessment tests, neuroimaging and clinical data (Sternberg et al., 2017). This at least partly indicates that iron can act as a biomarker for AD.

## Iron-Targeting Treatment Strategies

Metal-chelating agents can bind metal ions to the inside of a chelating agent through the strong binding action of a chelating agent molecule with a metal ion to become a stable compound with a larger molecular weight, thereby preventing metal ions from acting. Since iron overload plays an important role in the occurrence and development of AD, the use of metal chelators to reduce excessive iron in certain areas of the brain of AD patients, to achieve the strategy of relieving or even treating AD, has received an increased amount of attention. For a metal chelator to effectively exert chelation, it must have the following characteristics: (1) easy to penetrate the cell membrane and blood-brain barrier (BBB); (2) target iron-enriched areas without depleting transferrin-bound iron from plasma; (3) remove chelated iron from iron accumulation sites or transfer it to other biological proteins, such as circulating transferrin; and (4) no side effects or minor side effects on the body (Boddaert et al., 2007).

### Clioquinol

Clioquinol (CQ) has the chemical name 5-chloro-7-iodo-8-hydroxyquinoline and is an effective metal (iron, copper, and zinc) chelating agent. Studies have found that treatment of animal models of AD with CQ can reduce the deposition of amyloid in the brain and improve memory impairment (Cherny et al., 2001; Grossi et al., 2009). The probable cause of this phenomenon is that the high binding affinity of CQ to iron, zinc and copper ions allows it to competitively seize these metals from Aβ and prevent aggregation of Aβ (Opazo et al., 2006). Another study showed that 15-month-old APP Tg2576AD mice treated with CQ demonstrated a significantly reduced number and sizes of SPs in the brain compared with those of the sham-treated littermates (Cherny et al., 2001). Simultaneously, an *in vivo* experiment also found that, compared with the control group, CQ reduced the expression of APP by inhibiting the expression of β-secretase (BACE1) and γ-secretase (PS1) in the brains of APP/PS1 double transgenic mice (Wang et al., 2012). In coincidence with the results in animal models of AD, a small phase 2 clinical trial, it was also found that, compared with the control group, patients with moderately severe AD after oral CQ demonstrated slower decline in cognitive function and decreased Aβ42 levels in cerebrospinal fluid (Ritchie et al., 2003). However, it still lacks of the direct evidences supporting that CQ rescues the AD-like phenotypes via targeting iron *in vivo*. As is well known that tau deficiency induced age-dependent iron accumulation can be prevented by oral treatment with CQ (Lei et al., 2012, 2015). Moreover, CQ treatment can effectively prevent an iron-synuclein interaction in hA53T transgenic mice (Billings et al., 2016), as well as reverse the Fe^3+^-induced fibrin formation *in vitro* (Pretorius et al., 2013). Importantly, the formation of Aβ40 and Aβ42 aggregates in the presence of Fe^3+^ and Cu^2+^ were investigated, the study demonstrated that Fe^3+^, but not Cu^2+^, promotes the aggregation of Aβ40 and Aβ42, and CQ significantly reduces the Fe^3+^-induced Aβ42 aggregation (Tahmasebinia and Emadi, 2017). These researches provide the evidence that the anti-AD ability of CQ may, at least in part, via targeting iron and, surely, the underlying mechanism need to be further elucidated, Although it is now thought that CQ is toxic to the body, it at least opens up a promising direction for us.

### Desferoxamine, Deferasirox and Deferiprone

Desferoxamine (DFO) is a well-proven iron chelator that inhibits the toxicity of iron or aluminum and the ROS that it induces on the body. It was first thought that DFO was a chelating agent for aluminum ions, and aluminum was thought to be an independent factor that increased the risk of AD (Campbell and Bondy, 2000). The results of Crapper McLachlan et al. (1991) showed that the degree of decline in the daily living abilities of AD patients given intramuscular injections of DFO was alleviated compared with AD patients given a placebo. *In vitro* experiments showed that DFO can inhibit the formation of β-sheets of Aβ1-42 and dissolve preformed plaque-like amyloid plaques (House et al., 2004). There are also studies that have shown that DFO can inhibit the translation of APP mRNA and the expression of APP whole protein and reduce the secretion of Aβ peptides (Rogers et al., 2002; Morse et al., 2004). Our research revealed that nasal feeding of DFO can reverse iron-induced memory deficits in AD mice and inhibit the formation of APP (Guo et al., 2013b). In addition to its effect on APP, DFO also influences the phosphorylation of tau protein. Fine et al. (2012) have found that DFO has the ability to phosphorylate GSK-3β, which in turn reduces the level of phosphorylated tau, but the mechanism of its inhibition of tau protein phosphorylation is not yet clear. Although DFO can inhibit the phosphorylation of tau protein in the brain of AD model mice, the effect of DFO on the phosphorylation of tau protein has not been determined in the presence of iron. Our previous experiments, which involved feeding APP/PS1 transgenic mice high concentrations of iron and then nasally administered DFO to the mice, showed that intranasal DFO treatment inhibited iron-induced tau phosphorylation through the CDK5 and GSK-3β pathways (Guo et al., 2013a). We also found that DFO can also attenuate synaptic loss in the brain of APP/PS1 transgenic mice through the P38/HIF-1α pathway (Guo et al., 2015).

Although DFO has achieved certain results in a variety of experimental mouse models and has been approved by the Federal Drug Administration (FDA) for the treatment of iron overload disease, there are still many problems in the clinical application of DFO. First, the bioavailability of DFO is poor, and the molecular size and hydrophilicity of DFO prevent it from freely crossing the BBB, reducing its availability in the central nervous system; Second, DFO cannot be taken orally and must be given by injection. The time of a single injection is long (up to 10 h) and the frequency of injections is high (5–7 times per week), resulting in low patient compliance (Crielaard et al., 2017); Again, there are many side effects, including neurotoxicity after long-term treatment and systemic metal ion depletion accompanied by anemia (Cuajungco et al., 2000), gastrointestinal malabsorption and rapid degradation (May and Bulman, 1983).

Deferasirox is the first FDA-approved oral iron repellent that can be routinely used. Its chemical name is 4-[3,5-bis(2-hydroxyphenyl)-1,2,4-tris Oxazol-1-yl]benzoic acid, and it is commonly used as a treatment for patients with thalassemia iron overload. Deferasirox’s ability to bind iron is limited. This drug can only bind part of the iron and supply it to the extracellular and intracellular iron receptors. It is not easy to induce iron deficiency, but the efficacy of reducing iron accumulation is also relatively low. At present, studies have found that deferasirox plays a role in reducing brain iron accumulation. However, some studies have shown that deferasirox does not reduce brain iron accumulation or reduce iron toxicity in the brain (Sripetchwandee et al., 2016). This may be because deferasirox does not easily pass through the BBB, and its ability to bind iron is also weak. Three molecules of deferasirox are required for each binding molecule of iron, and thus higher doses are required.

The chemical name of deferiprone is 3-hydroxy-l,2-dimethyl-4-(lH)-pyridone. Like deferasirox, deferiprone is also approved for the treatment of patients with thalassemia iron overload. Deferiprone can bind almost all of the iron in the body to make it unable to further induce the production of ROS, and can also supply the bound iron to the iron receptors inside and outside the cell. Since deferiprone has a high iron-binding capacity, two molecules of deferiprone are required for each binding of one molecule of iron, and thus iron aggregation can be effectively reduced. Studies have found that deferiprone and DFO exert protective effects by reducing the rate of BBB disintegration, reducing brain iron accumulation and brain mitochondrial dysfunction in the presence of iron overload (Sripetchwandee et al., 2016). Although both deferiprone and DFO exert a protective effect, deferiprone can achieve greater advantages by oral administration.

Currently, DFO, deferasirox and deferiprone are recommended as iron overloaded first-line iron chelators, but they all have certain side effects, including allergic reactions, liver and renal dysfunction, and neuronal hearing loss (Borgna-Pignatti and Marsella, 2015). This requires further development of iron chelation therapy.

### α-lipoic Acid

α-lipoic acid (LA) is a small molecule compound that can be naturally synthesized in mammals. It is a coenzyme that exists in mitochondria. Its structure contains hydroxyl and disulfide bonds, so it has both fat-soluble and water-soluble properties, and it easily crosses the BBB. The study found that, after 60 min of intravenous injection of LA in mice, the traces of LA were observed in the mouse cerebral cortex (Panigrahi et al., 1996). After a continuous injection over 7–14 days, the presence of LA was detected in multiple parts of the mouse brain (Arivazhagan et al., 2002). Surprisingly, however, when a certain amount of LA was administered to mice daily by gavage, LA accumulation was not detected in the mouse brain after a period of time (Chng et al., 2009). The most likely reason for this is that LA is quickly reduced to dihydro-lipoic acid (DHLA) in the stomach and is transported through the blood circulation to tissues throughout the body to participate in metabolism and is eventually excreted in urine.

In Europe, LA has been used as a therapeutic drug for more than 50 years, and it is mainly used for the treatment of diabetic polyneuropathy. Later, in a clinical study, it was found that the decline in cognitive abilities of 129 patients who may have been suffering from AD was effectively alleviated after LA treatment for some time (Fava et al., 2013). However, the sample size of this study was relatively small, and there was no randomized sample. The study subjects were likely AD patients and were not confirmed by neuropathology. Therefore, the results were not very convincing. Several studies have shown that supplementation with LA can increase the activity of acetylcholine transferase (ChAT) in the brains of rodents and relieve their cognitive impairment (de Freitas, 2010; Dwivedi et al., 2014). The activation of ChAT positively regulates the content of acetylcholine in the brain, and the decreased expression of acetylcholine is closely related to the impairment of cognitive function in AD. Therefore, LA may increase the expression of acetylcholine by activating ChAT and improve the cognitive function of AD patients. At the same time, studies have shown that the activation of ChAT is also beneficial to the neurogenesis of cholinergic neurons (Park et al., 2013), which further demonstrates that LA may improve the severity of central nervous system diseases. A large number of studies have shown that LA can reduce the release of IL-1β, IL-6, and TNFα by inhibiting the activity of the NF-κB and MAPK signaling pathways (Bierhaus et al., 1997; Wong et al., 2001; Zhang and Frei, 2001). Simultaneously, LA also inhibits the infiltration of inflammatory cells into the central nervous system and downregulates the expression of vascular cell adhesion molecule-1 (Kunt et al., 1999; Guo et al., 2016). In addition to its ability to chelate metal ions, LA can also play a role in anti-inflammation, antioxidant and activated glucose uptake and utilization. So far, no serious side effects of LA treatment have been observed, which also indicates that LA may become a trend for clinical drug development for AD treatment.

*In vitro* studies have shown that LA can bind with metal ions, such as Cu^2+^, Zn^2+^, and DHLA, and can form complexes with Cu^2+^, Zn^2+^, Pb^2+^, Hg^2+^, and Fe^3+^, thereby exerting metal chelation (Ou et al., 1995). Treatment of lens epithelial cells with LA causes a significant decrease in iron uptake rates and dynamic iron pools within the cells (Goralska et al., 2003). These results suggested that LA not only reduces the level of iron that enters the cell but also reduces the dynamic iron pool within the cell by increasing iron stores. *In vivo* studies have shown that feeding of LA for 2 weeks in aged rats can reduce iron accumulation in the age-related cortical regions (Suh et al., 2005). The results of Fonte et al. (2001) showed that LA enhances the solubility of Aβ in the frontal cortex of APP-overexpressing transgenic mice, confirming that LA, like other metal ion chelators, can successfully resolubilize Aβ and reduce amyloid deposition in the brains of AD patients. However, in another study, learning and memory retention of LA-treated AD mice was significantly improved, but there was no significant changes in soluble or insoluble Aβ levels in the brain (Quinn et al., 2007). Although the metal chelation of LA has been proven by many experimental results, there is controversy as to whether it can inhibit the deposition of Aβ and increase the solubility of Aβ by chelating iron. Most recently, our group also found that chronic treatment with LA effectively inhibited tau hyperphosphorylation and alleviated neuronal degeneration and abnormal behavior in P301S tau transgenic mice; the improvement is accompanied by alleviation of oxidative stress, inflammation and ferroptosis in the brains of transgenic mice (Zhang et al., 2018).

### Lactoferrin

Lactoferrin (LF) is a non-heme iron-binding glycoprotein with a molecular weight of 80 kDa that is widely present in various secretions, such as milk, saliva, and urine. Because its amino acid sequence is 60% identical to that of transferrin, it is classified as a transferrin family member (Metz-Boutigue et al., 1984). LF is composed of two spherical leaves folded from a polypeptide chain of 703 amino acids. Each molecule can bind reversibly with two iron, zinc, copper or other metal ions. The binding site is located on the two globular domains of the protein. The affinity of LF for iron is 300 times that of transferrin, and the affinity is further enhanced in weakly acidic environments, which may be related to the transfer of iron from transferrin to LF when inflammation occurs (Dhennin-Duthille et al., 2000). It has also been found that, in the central nervous system, activated microglia can also generate and release LF (Xu et al., 2017). The results of immunohistochemistry studies showed that LF accumulated in the brain tissue of patients with neurodegenerative diseases. Further studies have found abnormal LF content and distribution in the brains of AD patients (Brown et al., 1985), with a large amount of LF deposition in the SP- and NFT-enriched areas (Valverde et al., 1990). Wang et al. (2010) detected LF deposition in the brains of AD model mice, but no LF deposition was found in wild mice, and the formation of SPs preceded LF. Further studies showed that LF deposition was localized in SPs and amyloid lesions. LF deposition increased with the age of transgenic mice, but after 18 months of age, most SPs showed a decrease in LF positives (Wang et al., 2010). Using laser confocal technology in AD transgenic mice to colocalize LF and Aβ in the brain, it was found that LF is expressed on Aβ, Aβ formation precedes deposition of LF, and Aβ plaques develop as the age of the rat increases and as both the size and number of LF accumulations increase, indicating that there is a close relationship between LF and AD (van de Looij et al., 2014). In combination with other biological functions of LF, such as its participation in metal ion metabolism and the regulation of cell proliferation and apoptosis, we hypothesize that LF may delay the occurrence of active AD.

With the presence of LF receptors (LFR) on the blood membrane of BBB vascular endothelial cells, exogenous LF can easily cross the BBB; thus, in recent years, LF has been widely used as a carrier for drug targeting of the brain. Interestingly, Kamalinia et al. (2013) used the PC12 cell line to demonstrate that lactoferrin-DFO conjugates are able to interfere with apoptosis. The expression levels of autophagy markers, including Atg7, Atg12-Atg5 and LC3-II/LC3-I, increased, and the LF-conjugated peritoneal cavity mainly affected the expression levels of Capsase-3, PARP, Bax and bcl-2. Furthermore, intraperitoneal injection of LF conjugates can significantly improve the learning abilities of AD rats and reduce Aβ (Kamalinia et al., 2013), which provides a theoretical basis for the use of LF in the treatment of neurodegenerative diseases.

Therefore, the use of exogenous LF as a therapeutic agent has been investigated to identify its roles in AD. Our group was the first to investigate whether exogenous LF administration could stimulate the non-amyloidogenic processing of APP and α-secretase catalytic activity and expression, which consequently reduce Aβ deposition and ameliorate cognitive decline in AD model mice (Guo et al., 2017). We also addressed the molecular mechanisms by which LF modulates APP processing. In fact, it has been reported in the literature that the function of human LF is similar to that of the iron chelator DFO, and it can also induce the neuroprotective effect of hypoxia-inducible factor (HIF-1α) expression under hypoxic conditions (Kawamata et al., 1993). Specifically, our results suggested that LF can enhance the α-secretase-dependent APP process through the ERK1/2-CREB and HIF-1α pathways *in vitro* and *in vivo* (Guo et al., 2017), once again proving that LF plays an active role in the treatment of AD. Consistent with previous observations, Aizawa et al. (2017) recently reported that the binding of LF to low-density lipoprotein receptor-associated protein-1 (LRP1) leads to the activation of the AMP-activated protein kinase signaling pathway, which in turn, promotes cell autophagy. This shows that LF may regulate the production of Aβ and NFTs through anti-inflammation, regulation of immunity, chelation of metal ions, regulation of cell autophagy, and may ultimately affect the occurrence and development of AD.

## Conclusion

The pathogenesis of AD has been studied for decades, and although it is clear that SPs formed by Aβ deposition and NFTs formed by hyperphosphorylation of tau protein are two major pathological features of AD pathogenesis, there are many opinions about the inducing factors of SPs and NFTs that have not been clearly clarified yet. In light of the complex causes of AD, the study of its pathogenesis from multiple perspectives has been accepted by the public and has achieved good results. The study of the pathogenesis of AD using iron as a target has become a research hotspot in recent years. The use of iron chelators to chelate excess iron in the brains of AD patients has also become a new strategy for the treatment of AD. However, unlike other tissues and organs, there is a BBB in the brain that has strict specificity for the entry of drugs. Therefore, finding an iron chelator that easily crosses the BBB and that can function effectively must be sought from the body’s self-synthesizing substances. There are many proteins or molecules that bind to iron in the body, and LA and LF are among these candidate drugs. *In vitro* and *in vivo* experiments have confirmed that direct administration of LA and LF can alleviate the symptoms of AD (Guo et al., 2017; Zhang et al., 2018), but the relevant mechanism of action on them has not been clarified, and further studies are needed. At the same time, LF-based nano-drug molecules are also under study. This drug molecule attaches to the LF molecule and can easily cross the BBB, thereby exerting a therapeutic effect on lesions and achieving better therapeutic effect. Future research will also use iron as an opportunity to study the mechanism of the occurrence and development of AD from the iron steady state to more fully explain the causes of AD.

## Author Contributions

J-LL and Y-GF wrote the manuscript. Z-SY, Z-YW, and CG approved and revised the final manuscript. All the authors have read and approved the final manuscript.

## Conflict of Interest Statement

The authors declare that the research was conducted in the absence of any commercial or financial relationships that could be construed as a potential conflict of interest.
